# Depolarization and repolarization dynamics after His‐bundle pacing: Comparison with right ventricular pacing and native ventricular conduction

**DOI:** 10.1111/anec.12991

**Published:** 2022-07-08

**Authors:** Satoshi Yanagisawa, Yasuya Inden, Ryo Watanabe, Naoki Tsurumi, Noriyuki Suzuki, Toshifumi Nakagomi, Masafumi Shimojo, Takashi Okajima, Shuro Riku, Koichi Furui, Kazumasa Suga, Rei Shibata, Toyoaki Murohara

**Affiliations:** ^1^ Department of Advanced Cardiovascular Therapeutics Nagoya University Graduate School of Medicine Nagoya Japan; ^2^ Department of Cardiology Nagoya University Graduate School of Medicine Nagoya Japan

**Keywords:** His‐bundle pacing, QT interval, repolarization, right ventricular pacing, T‐peak to T‐end

## Abstract

**Background:**

The current study aimed to evaluate changes in electrical depolarization and repolarization parameters after His‐bundle pacing (HBP) compared with right ventricular pacing (RVP) and its association with ventricular arrhythmia (VA).

**Methods:**

Forty‐one patients (13 with HBP, 14 with RVP, and 14 controls [AAI mode]) were evaluated. After continuous pacing algorithm, QRS duration, QT interval, QTc, JT interval, T‐peak to T‐end (Tpe), and Tpe/QT ratio were measured on electrocardiography at baseline and 1 week, 1 month, and 6 months postoperatively. We investigated VA occurrence and adverse events after implantation.

**Results:**

At 6 months, QRS duration was significantly shorter in the HBP (121.6 ± 15.6 ms) than in the RVP (150.1 ± 14.9 ms) group. The QT intervals were lower in the HBP (424.0 ± 40.9 ms) and control (405.9 ± 23.0 ms) groups than in the RVP (453.0 ± 40.2 ms) group. The Tpe and Tpe/QT ratios at 6 months differed significantly between the HBP and RVP groups (Tpe, 69.8 ± 19.7 ms vs 87.4 ± 11.9 ms and Tpe/QT, 0.16 ± 0.03 vs 0.19 ± 0.02, respectively). The Tpe and Tpe/QT ratios were similarly shortened in the HBP and control groups. VA occurred less frequently in the HBP (15%) and control (7.1%) groups than in the RVP (50%) group (*p* = 0.020). The non‐RVP group showed significantly lower rates of VA and major adverse events than the RVP group. Patients with VA demonstrated significantly longer QRS duration, QT interval, Tpe, and Tpe/QT at 6 months than those without VA.

**Conclusion:**

HBP showed better depolarization and repolarization stability than RVP.

## Graphical abstract

Copyright Transfer Agreement (CTA) form

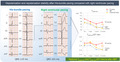

## 1 INTRODUCTION

His‐bundle pacing (HBP) achieves physiological conduction to the ventricle, narrow QRS duration, and ventricular activation synchronization (Deshmukh et al., 2000; Hasumi et al., 2019; Kataoka et al., 2019). Previous studies demonstrated the efficacy of HBP at reducing hospitalization due to heart failure (HF) and mortality compared with right ventricular pacing (RVP) (Abdelrahman et al., 2018; Vijayaraman, Naperkowski, et al., 2018).

HBP may have another possible advantage in terms of depolarization and repolarization stability versus RVP; longer QRS duration and prolonged QT interval derived from RVP are associated with a high risk of ventricular arrhythmia (VA) and mortality (Cho et al., 2016; Del‐Carpio Munoz et al., 2017). An abrupt change in the activation sequence of the ventricle after RVP induces heterogeneous ventricular repolarization in the acute phase, while structural remodeling of the ventricle increases the repolarization instability and risk of proarrhythmic effects during the chronic phase (Itoh et al., 2013; Marrus et al., 2012; Wecke et al., 2005, 2007). In contrast, few studies have examined the effects of HBP on depolarization and repolarization abnormalities.

This study aimed to evaluate changes in depolarization and repolarization parameters on electrocardiography (ECG) after HBP versus RVP and assess its association with VA occurrence.

## 2 METHODS

### 2.1 Study population

The study population was retrospectively recruited from the Nagoya University Hospital database. Patients who underwent pacemaker implantation due to atrioventricular block between January 2016 and June 2021 as continuous HBP and RVP and who could be examined by 3‐min ECG at baseline, 1 week, 1 month, and 6 months postoperative were included. We also included a control group of patients who underwent pacemaker implantation for sick sinus syndrome during the same study period. These patients were followed up in atrial demand pacing (AAI) pacing mode and intrinsic ventricular rhythm (QRS duration <130 ms). The present study excluded patients with a baseline left ventricular ejection fraction (LVEF) <40%. All patients undergoing pacemaker implantation were selected according to recent guidelines (Nogami et al., 2021, 2022). Forty‐one patients (13 with HBP, 14 with RVP, and 14 controls) were included. All patients received continuous ventricular pacing (≥90%) using the same pacing algorithm without any adverse events or invasive procedures during the study period. Their medications were continued throughout the study period without changes or additions. All patients provided written informed consent prior to implantation. The study protocol was approved by the Institutional Ethics Committee. This study was performed in accordance with the principles of the Declaration of Helsinki.

### 2.2 Device implantation

The details of HBP implantation have been described previously (Yanagisawa et al., 2019). First, the atrial lead was inserted into the right ventricle (RV) as backup ventricular pacing. The pacing lead (Select Secure 3830; Medtronic Inc.) was advanced to fix the His‐bundle region using a specific sheath (C315His; Medtronic, Inc.). A unipolar electrogram recording from the lead tip was displayed using both a Medtronic pacing system analyzer and an electrophysiologic recording system at a sweep speed of 100 mm/s. Pacing was applied from 5.0 V at a 1.0 ms width to the minimum output while simultaneously checking pacing morphology on 12‐lead surface ECG and local electrogram to facilitate His‐bundle capture or RV capture when the His‐bundle electrogram could be identified in the distal tip of the His‐bundle lead. The lead was then screwed into position using clockwise rotations with maintained contact. The pacing threshold of His‐bundle capture and RV capture was tested again, and 1:1 His‐ventricular conduction by high‐rate pacing was confirmed. Generally, a pacing threshold for His‐bundle capture of <2.5 V at a 1.0‐ms width was considered acceptable. Pacing morphology, which was classified as selective HBP, non‐selective HBP, and RVP, was defined according to the criteria proposed by a multicenter collaborative working group (Vijayaraman, Dandamudi, et al., 2018). Non‐selective HBP was defined as the capture of the His‐bundle and local ventricular myocardium and the involvement of two distinct pacing thresholds for His‐bundle capture and RV capture. There was no isoelectric interval duration between the pacing stimulus and QRS onset on the electrogram in the presence of a pseudo‐delta wave in non‐selective HBP. Selective HBP is defined as ventricular activation exclusively over the His‐Purkinje conduction system, with only capture of the tissue of the His‐bundle. To achieve stable HBP, we paid special attention to a lower local ventricular threshold as a backup for permanent HBP (Sato et al., 2021). In cases of a higher pacing threshold or non‐capture of the His‐bundle after repetitive mapping and fixation, the pacing lead was fixed to the RV septum. Thereafter, the atrial lead was fixed to the right atrial appendage. The atrial and ventricular leads for conventional pacemakers were placed at the right atrial appendage and the RV septum, respectively.

### 2.3 ECG assessment

QRS duration, QT interval, QTc (B), J‐point to T‐end (JT) interval, T‐peak to T‐end (Tpe), and Tpe/QT ratio were manually measured using 3‐min ECG before implantation and 1 week, 1 month, and 6 months postoperatively. The ECG was digitally recorded at a paper speed of 25 mm/s and a scale of 10 mm/mV (Cardio Star, FCP‐7541; Fukuda Denshi). All intervals and parameters were measured at 200% magnification of the ECG using a digital caliper. We analyzed the QRS‐T complex in the last phase of the 3‐min ECG measurement over ≥3 consecutive stable rhythm beats. The QT interval was measured using the tangent method. We determined the maximum duration of the QT interval among all leads that could demonstrate a steep T wave with a clear offset. QTc (B) is the corrected QT interval determined by heart rate using Bazett's formula. The JT interval was measured as the time from the J‐point to the end of the T wave after exclusion of the QRS component. The Tpe was measured from the peak to the end of the T wave.

### 2.4 Follow‐up

All patients were followed up at 1 and 6 months postoperatively and every 6–8 months thereafter in the outpatient clinic. Any subsequent VA events, including sustained ventricular tachycardia (>30 s) and non‐sustained ventricular tachycardia (>5 s), were collected from the device interrogation. Adverse events, including all‐cause death, HF hospitalization, and VA events during the follow‐up period, were assessed via review of the patients' medical records. In all patients, transthoracic echocardiography was performed before device implantation. Follow‐up echocardiography at 6 months was also performed if applicable.

### 2.5 Statistical analysis

Continuous variables are expressed as mean ± standard deviation, while categorical variables are presented as number (%). Student's *t*‐test was used to compare continuous variables, while the chi‐squared test or Fisher's exact test was used to compare categorical variables. Differences in the baseline characteristics between more than two different groups were analyzed using a one‐way analysis of variance and the chi‐squared test, as appropriate. Repeated measures of analysis of variance with multiple pacing modes were used to evaluate significant differences in the mean values over time in the same patients. A post hoc analysis of the ECG parameters within the pacing group from baseline to 6 months and those at the specific follow‐up time points between the different pacing modes was performed using Bonferroni correction for multiple comparisons. Event‐free rates were calculated using Kaplan–Meier survival curve analysis with the log‐rank test. Statistical significance was set at *p* < .05. All analyses were performed using IBM SPSS Statistics for Windows version 25.0 (SPSS Inc).

## 3 RESULTS

### 3.1 Baseline characteristics

The baseline characteristics of the different pacing modes are presented in Table 1. There were no significant intergroup differences in age, sex, history of HF, or atrial fibrillation. All patients in the HBP group continued non‐selective HBP, except for one patient with selective HBP. Two patients had a completed right bundle branch block before HBP implantation that was corrected by HBP during the study evaluation period thereafter. Eight patients underwent RVP after HBP failure, while six underwent primary RVP because the HBP system was not available. The mean LVEF was similar in the HBP (67.4 ± 4.8%), RVP (61.5 ± 9.3%), and control groups (63.3 ± 4.5%) (*p* = 0.075). The B‐type natriuretic peptide levels were significantly higher in the RVP group (286.0 ± 307.3 pg/dl) than in the HBP (72.4 ± 80.1 pg/dl) and control (115.4 ± 74.6 pg/dl) groups. Six patients had structural heart disease (valvular heart disease and old myocardial infarction) in the HBP (*n* = 2), RVP (*n* = 3), and control (*n* = 1) groups. None of the patients had a history of clinical VA requiring additional treatment before the procedure. At 6‐month post‐implantation, although the mean LVEF was preserved within the normal limit in the HBP group (67.7 ± 4.4%), it decreased slightly from 61.5 ± 9.3% to 58.4 ± 9.4% after 6 months in the RVP group.

**TABLE 1:** Comparison of baseline characteristics among the different pacing groups

Parameters	HBP (*n* = 13)	RV pacing (*n* = 14)	Control (AAI mode) (*n* = 14)	*p*‐value
Age, year	75.6 ± 8.0	77.9 ± 9.3	74.8 ± 5.6	.580
Male sex (%)	5 (39)	8 (57)	5 (36)	.464
Body mass index (kg/m^2^)	23.6 ± 4.6	23.3 ± 4.0	24.7 ± 5.4	.693
QRS duration before implantation (ms)	112.2 ± 30.6	134.4 ± 34.2	106.1 ± 15.7^c^	.028
Anti‐arrhythmic drugs, Class III	0 (0%)	1 (7.1%)	2 (14%)	.363
History of atrial fibrillation	3 (23%)	6 (43%)	9 (64%)	.097
History of heart failure	1 (7.7%)	4 (29%)	1 (7.1%)	.191
Structural heart disease	2 (15%)	3 (21%)	1 (7.1%)	.562
Previous temporal RV pacing	1 (7.7%)	2 (14%)	0 (0%)	.348
Echocardiographic data				
Left atrial diameter (mm)	36.4 ± 11.0	41.6 ± 5.3	43.9 ± 6.5	.051
LVEF (%)	67.4 ± 4.8	61.5 ± 9.3	63.3 ± 4.5	.075
LVEDD (mm)	46.0 ± 3.6	48.9 ± 4.2	46.3 ± 8.7	.391
LVESD (mm)	28.1 ± 2.9	31.9 ± 5.9	29.3 ± 5.4	.141
LVEF after 6 months (%)	67.7 ± 4.4^a^	58.4 ± 9.4^a^	n/a	.069
LVEDD after 6 months (mm)	45.9 ± 6.3^a^	48.7 ± 5.0^a^	n/a	.269
LVESD after 6 months (mm)	28.4 ± 4.9^a^	32.9 ± 5.1^a^	n/a	.060
B‐type natriuretic peptide levels (pg/dl)	72.4 ± 80.1^b^	286.0 ± 307.3	115.4 ± 74.6	.013

*Note*: The data are presented as *n* (%) and means ± standard deviations.

Differences in the baseline characteristics among the groups were analyzed using a one‐way analysis of variance, and the chi‐squared test. A post hoc analysis of the comparison of the parameters between the different pacing modes was performed using a Bonferroni correction for the multiple comparisons.

Abbreviations: AF, atrial fibrillation; HBP, his‐bundle pacing; LVEDD, left ventricular end‐diastolic diameter; LVEF, left ventricular ejection fraction; LVESD, left ventricular end‐systolic diameter; RV, right ventricle.

^a^ Data were available for a total of 10 and 10 patients in the HBP and RV pacing groups, respectively.

^b^ *p* < .05 HBP vs RV pacing.

^c^ *p* < .05 control vs RV pacing.

At baseline, QT and QTc (B) intervals were relatively higher in the RVP group than in the HBP and control groups (QT interval: 485.5 ± 59.2 ms, 524.3 ± 106.3 ms, and 470.1 ± 50.6 ms; QTc (B) interval: 442.1 ± 38.9 ms, 472.5 ± 62.8 ms, and 453.5 ± 51.6 ms in the HBP, RVP, and control groups, respectively).

### 3.2 Follow‐up assessment of ECG parameters after implantation

**FIGURE 1:**
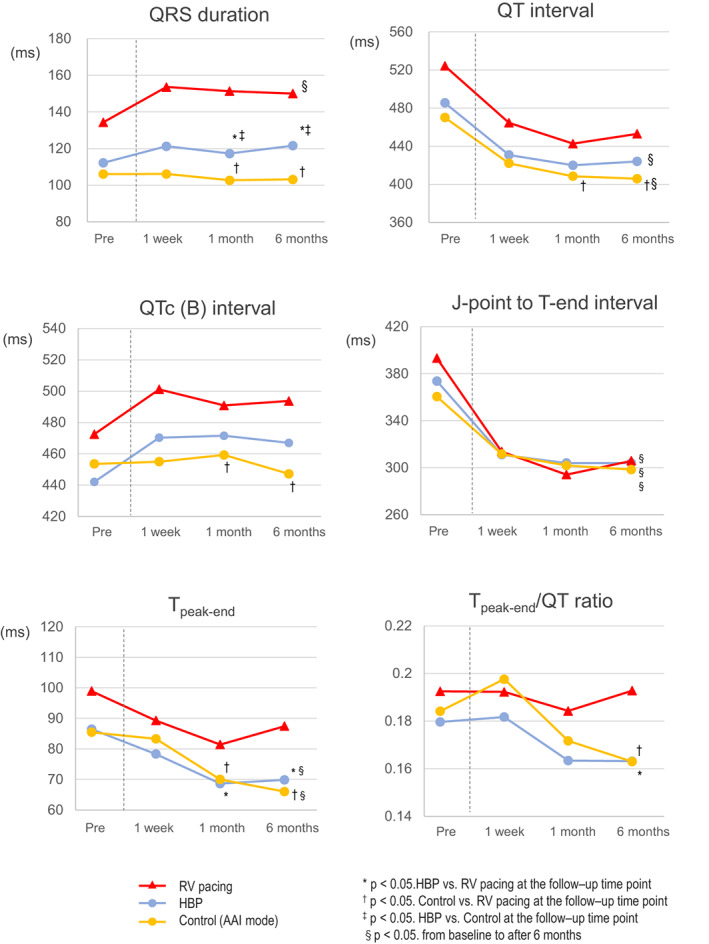
Changes in electrogram parameters before versus after implantation by pacing group. Repeated measures of analysis of variance with multiple pacing modes were used to evaluate significant differences in the mean values over time in the same patients. A post hoc analysis of the electrocardiography parameters within the pacing group from baseline to 6 months and those at the specific follow‐up time points between the different pacing modes was performed using Bonferroni correction for multiple comparisons. HBP, his‐bundle pacing; QTc (B), corrected QT interval using Bazett's formula; RV, right ventricle. *p < .05, HBP vs RV pacing. ^†^ *p* < .05, control vs RV pacing. ^‡^ *p* < .05, HBP vs control. ^§^ *p* < .05, from baseline to after 6 months

After 6 months, QRS duration was significantly shorter in the HBP versus RVP group (121.6 ± 15.6 ms vs 150.1 ± 14.9 ms), and the difference was maintained following implantation (Figure 1, Table 2). The QT interval significantly improved after 6 months in the HBP (424.0 ± 40.9 ms) and control groups (405.9 ± 23.0 ms) than in the RVP (453.0 ± 40.2 ms). The QTc (B) at 6 months was lower in the HBP (466.9 ± 30.3 ms) and control (447.2 ± 27.1 ms) groups than in the RVP group (493.7 ± 40.1 ms). The JT interval significantly decreased 6 months after baseline in the HBP and RVP groups. The Tpe was significantly reduced in the HBP group from baseline to 6 months (from 86.5 ± 12.5 ms to 69.8 ± 19.7 ms, respectively). There was a significant difference in Tpe at 6 months between the HBP and RVP groups (69.8 ± 19.7 ms vs 87.4 ± 11.9 ms, *p* < 0.05). The Tpe/QT ratio at 6 months was significantly lower in the HBP group than in the RVP group (0.16 ± 0.03 vs 0.19 ± 0.02, respectively; *p* < .05). In contrast, the Tpe and Tpe/QT ratios were similarly shortened in the HBP and control groups after 6 months.

**TABLE 2:** Changes in electrocardiogram parameters after device implantation

Parameters	Baseline	After 1 week	After 1 month	After 6 months	*p* for trend (overall)
QRS duration (ms)					
HBP	112.2 ± 30.6	121.3 ± 18.5	117.3 ± 14.8^a^ ^,^ ^b^	121.6 ± 15.6^a^ ^,^ ^b^	.400
RV pacing	134.4 ± 34.2	153.6 ± 13.4	151.4 ± 11.2	150.1 ± 14.9	.016
Control (AAI mode)	106.1 ± 15.7^c^	106.1 ± 16.5	102.7 ± 18.2^c^	103.1 ± 16.1^c^	.240
QT interval (ms)					
HBP	485.5 ± 59.2	430.8 ± 41.5	420.0 ± 24.5	424.0 ± 40.9^d^	<.001
RV pacing	524.3 ± 106.3	464.6 ± 38.5	442.7 ± 40.6	453.0 ± 40.2	.021
Control (AAI mode)	470.1 ± 50.6	422.0 ± 36.3	408.5 ± 22.4^c^	405.9 ± 23.0^c^ ^,^ ^d^	<.001
QTc (B), ms					
HBP	442.1 ± 38.9	470.3 ± 43.7	471.5 ± 29.4	466.9 ± 30.3	.078
RV pacing	472.5 ± 62.8	501.1 ± 31.6	490.9 ± 28.7	493.7 ± 40.1	.195
Control (AAI mode)	453.5 ± 51.6	454.9 ± 32.9	459.3 ± 32.3^c^	447.2 ± 27.1^c^	.664
J‐point to T‐end interval (ms)					
HBP	373.7 ± 54.4	311.1 ± 32.1	303.9 ± 21.4	303.9 ± 31.9^d^	<.001
RV pacing	393.2 ± 94.9	313.6 ± 31.2	294.1 ± 32.4	305.9 ± 29.5^d^	.003
Control (AAI mode)	360.4 ± 54.3	311.6 ± 38.6	301.9 ± 21.3	298.4 ± 22.8^d^	<.001
T‐peak to T‐end (ms)					
HBP	86.5 ± 12.5	78.3 ± 16.6	68.6 ± 11.8^a^	69.8 ± 19.7^a^ ^,^ ^d^	<.001
RV pacing	98.9 ± 22.7	89.3 ± 13.5	81.4 ± 10.7	87.4 ± 11.9	.051^a^
Control (AAI mode)	85.4 ± 21.3^c^	83.3 ± 10.9	70.0 ± 9.0^c^	66.0 ± 8.9^c^ ^,^ ^d^	<.001
T‐peak to T‐end/QT ratio					
HBP	0.18 ± 0.03	0.18 ± 0.03	0.16 ± 0.03	0.16 ± 0.03^a^	.117
RV pacing	0.19 ± 0.05	0.19 ± 0.03	0.18 ± 0.02	0.19 ± 0.02	.871
Control (AAI mode)	0.18 ± 0.05	0.20 ± 0.02	0.17 ± 0.02	0.16 ± 0.02^c^	.002
R‐R interval (ms)					
HBP	1237.0 ± 308.6	832.3 ± 137.5	758.8 ± 118.2	791.4 ± 102.7^d^	<.001
RV pacing	1323.1 ± 447.7	866.1 ± 128.3	847.4 ± 149.2	868.4 ± 117.9	<.001
Control (AAI mode)	1098.6 ± 310.6	880.6 ± 106.3	817.7 ± 114.9	844.0 ± 98.5	<.001

*Note*: The data are presented as means ± standard deviations.

To evaluate significant differences in mean values over time in the same patients, repeated measures of analysis of variance with multiple pacing modes were used as a multi‐way analysis of variance. A post hoc analysis of the comparison of the electrogram parameters within the pacing group from baseline to 6 months and those at the specific follow‐up time points between the different pacing modes was performed using a Bonferroni correction for the multiple comparisons. The overall changes in electrogram parameter between the different pacing modes during the follow‐up were compared by multiple comparisons using Bonferroni adjustment.

Abbreviations: HBP, his‐bundle pacing; RV, right ventricle.

^a^ *p* < .05 HBP vs RV pacing.

^b^ *p* < .05 HBP vs control.

^c^ *p* < .05 control vs RV pacing.

^d^ *p* < .05 from baseline to after 6 months.

### 3.3 Comparison of clinical outcomes after continuous pacing

**FIGURE 2:**
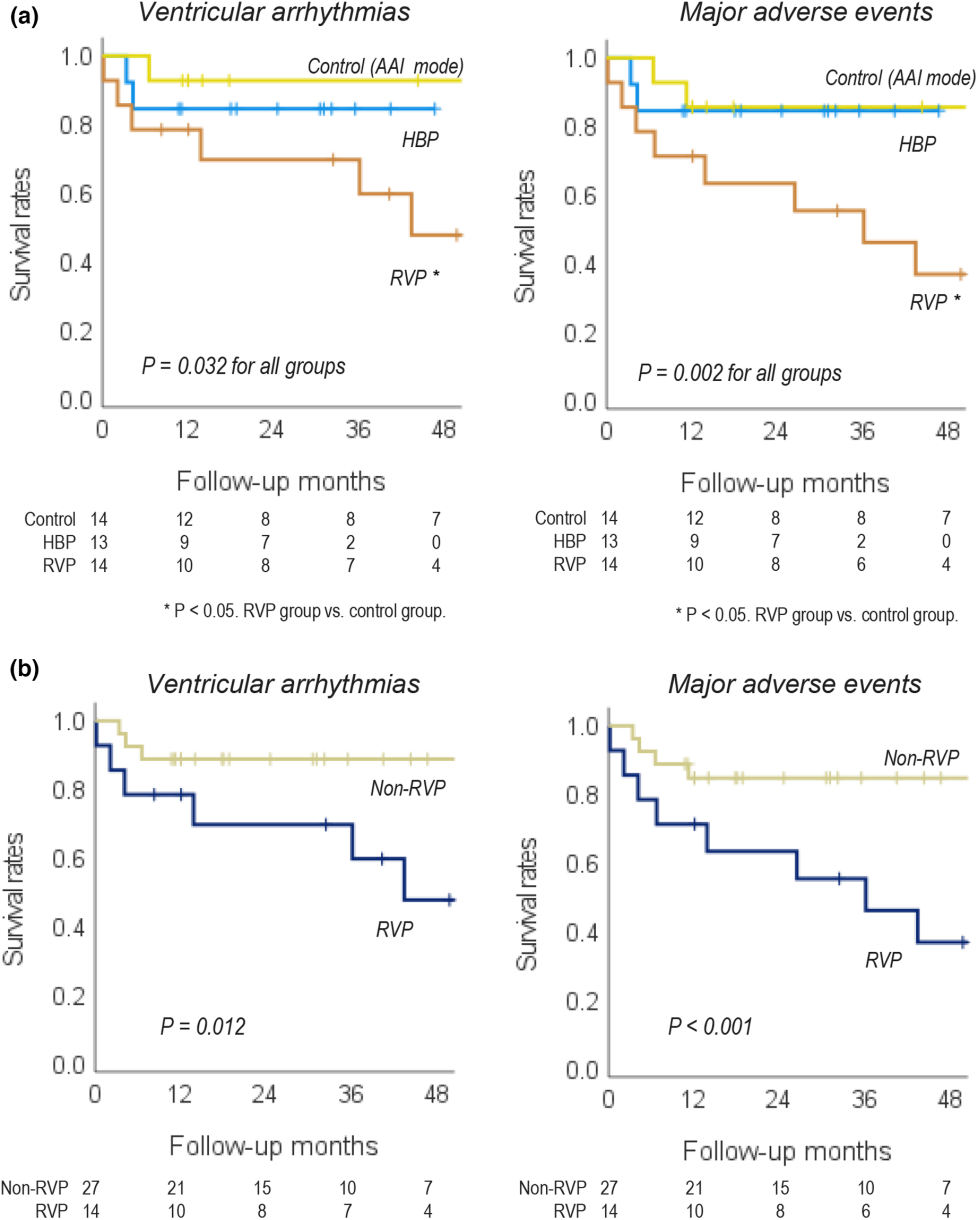
Comparison of ventricular arrhythmia occurrence and major adverse events (death, heart failure hospitalization, and ventricular arrhythmias) after implantation among the HBP, RVP, and control groups (a) and between the non‐RVP mode (HBP and control) versus RVP mode (b) treatments. HBP, non‐selective his‐bundle pacing; RVP, right ventricular pacing. *p < .05, RVP vs control group

During a mean follow‐up period of 31.6 months after implantation, 15% (*n* = 2), 50% (*n* = 7), and 7.1% (*n* = 1) patients from the HBP, RVP, and control groups had VA episodes, respectively (*p* = .020, Table 3). All VA events included non‐sustained ventricular tachycardias. The mean cycle length and number of the beats of the non‐sustained ventricular tachycardias were 356.6 ± 32.1 ms and 22.1 ± 9.8 beats, respectively. All ventricular tachycardias were monomorphic. Three patients died of non‐cardiac causes, and two more were hospitalized with HF after implantation. Major adverse events, including death, HF hospitalization, and VA, occurred in two (15%), 11 (79%), and two patients (14%) in the HBP, RVP, and control groups, respectively (*p* < .001; Table 3). The non‐RVP mode (HBP and control groups) had significantly fewer VA and major adverse events than the RVP mode (VA: three patients [11%] vs seven patients [50%], *p* = .017; adverse events: four patients [15%] vs 11 patients [79%], *p* < .001). Kaplan–Meier survival curve analyses demonstrated that patients in the RVP group had a significantly higher incidence of VA and adverse events during the follow‐up period than those in the non‐RVP pacing group (HBP and control groups) (Figure 2).

**TABLE 3:** Comparison of ventricular arrhythmias and major adverse events during the follow‐up period among the different pacing groups

Parameters	HBP (*n* = 13)	Control (AAI mode) (*n* = 14)	RVP (*n* = 14)	*p*‐value
All‐cause death	0 (0%)	1 (7.1%)	2 (14%)	.363
HF hospitalization	0 (0%)	0 (0%)	2 (14%)	.132
Ventricular arrhythmias	2 (15%)	1 (7.1%)^a^	7 (50%)	.020
Sustained ventricular arrythmias (>30 s)	0 (0%)	0 (0%)	0 (0%)	n/a
Non‐sustained ventricular tachycardias (>5 s)	3 (23%)	1 (7.1%)^a^	7 (50%)	.020
Total adverse events	2 (15%)^b^	2 (14%)^a^	11 (79%)	<.001
	Non‐RVP mode (HBP and control) (*n* = 27)		RVP mode (*n* = 14)	*p*‐value
Ventricular arrhythmias	3 (11%)		7 (50%)	.017
Total adverse events	4 (15%)		11 (79%)	<.001

*Note*: The data are presented as numbers (%). Differences in the outcomes among the groups were analyzed using the chi‐squared test.

Abbreviations: HBP, his‐bundle pacing; HF, heart failure; RVP, right ventricular pacing.

^a^ *p* < .05 control group vs RV pacing.

^b^ *p* < .05 HBP vs RV pacing.

A comparison of the baseline characteristics and examination results between the VA group (*n* = 10) and the non‐VA group (*n* = 31) is shown in Table 4. Patients with VA demonstrated significantly longer QRS duration (144.4 ± 18.0 ms vs 118.8 ± 23.8 ms, *p* = .003), QT interval (452.8 ± 32.6 ms vs 419.6 ± 39.1 ms, *p* = .020), Tpe (89.2 ± 14.7 ms vs 69.8 ± 14.5 ms, *p* = .001), and Tpe/QT ratio (0.20 ± 0.03 vs 0.17 ± 0.03, *p* = .002) at 6 months than those without VA. The VA group also included a higher prevalence of RVP patients than the non‐VA group (70% vs 23%, *p* = .017).

**TABLE 4:** Comparison of baseline characteristics and examination results between the VA group and non‐VA group among the three groups (the control, HBP, and RVP groups)

Parameters	VA group (*n* = 10)	Non‐VA group (*n* = 31)	*p*‐value
Age, year	76.8 ± 8.9	75.8 ± 7.4	.736
Male sex	8 (80%)	10 (32%)	.012*
QRS duration before implantation (ms)	126.6 ± 30.8	114.8 ± 29.6	.286
Anti‐arrhythmic drugs, Class III	0 (0%)	3 (10%)	.564
History of heart failure	2 (20%)	4 (13%)	.622
Echocardiographic data at baseline			
Left atrial diameter (mm)	40.7 ± 5.6	40.8 ± 9.1	.966
LVEF (%)	63.9 ± 9.2	64.0 ± 6.2	.975
LVEDD (mm)	48.3 ± 4.7	46.7 ± 6.4	.466
LVESD (mm)	31.1 ± 6.6	29.4 ± 4.6	.346
B‐type natriuretic peptide levels (pg/dl)	228.2 ± 286.2	138.0 ± 175.9	.237
Electrocardiogram parameters after 6 months			
QRS duration (ms)	144.4 ± 18.0	118.8 ± 23.8	.003*
QT interval (ms)	452.8 ± 32.6	419.6 ± 39.1	.020*
QTc (B) (ms)	486.6 ± 22.3	463.8 ± 40.1	.097
J‐point to T‐end interval (ms)	304.5 ± 26.7	302.1 ± 28.4	.815
T‐peak to T‐end (ms)	89.2 ± 14.7	69.8 ± 14.5	.001*
T‐peak to T‐end/QT ratio	0.20 ± 0.03	0.17 ± 0.03	.002*
RVP mode	7 (70%)	7 (23%)	.017*

*Note*: The data are presented as numbers (%) and means ± standard deviations.

The Student's *t*‐test was used to compare continuous variables, while chi‐squared test or Fisher's exact test was used to compare categorical variables.

Abbreviations: HBP, his‐bundle pacing; LVEDD, left ventricular end‐diastolic diameter; LVEF, left ventricular ejection fraction; LVESD, left ventricular end‐systolic diameter; RVP, right ventricular pacing; VA, ventricular arrhythmia.

^*^ *p* < 0.05. VA group vs non‐VA group.

### 3.4 Follow‐up pacing parameters in the HBP group

Pacing parameters at baseline and change in His‐capture threshold are shown in Table 5. Three patients had an increased His‐capture threshold of ≥1.0 V after implantation, but all patients continued HBP during the follow‐up period of 20.1 months. No patients required lead revision, HBP abandonment, or battery replacement after follow‐up.

**TABLE 5:** Pacing parameters in the HBP group

Parameters	HBP group (*n* = 13)
Type of HBP	
Selective HBP	1 (7.7%)
Non‐selective HBP	12 (92.3%)
R wave amplitude (mV)	3.9 ± 4.5
Bipolar pacing impedance (Ω)	513.0 ± 86.4
RV capture threshold (V at 1.0 ms)	1.24 ± 0.79
His‐capture threshold (V at 1.0 ms)	
Baseline	0.96 ± 0.56
1 week	0.65 ± 0.19
1 month	0.85 ± 0.55
6 months	0.13 ± 0.59
Increased His‐capture threshold of ≥1.0 V (from baseline to 6 months)	2 (15%)
Increased His‐capture threshold of ≥1.0 V (from baseline to the follow‐up end)	3 (23%)
HBP abandonment	0 (0%)
Lead revision	0 (0%)
Battery replacement	0 (0%)

*Note*: Values are presented as mean ± standard deviation or as n (%). HBP, His‐bundle pacing; RV, right ventricle.

## 4 DISCUSSION

The main findings of this study are as follows: (1) The HBP group showed a shorter QRS duration and QT interval than the RVP group after 6 months; (2) The Tpe and Tpe/QT ratios decreased throughout the evaluation period in the HBP and control groups and were significantly shorter than those in the RVP group; (3) After HBP implantation, reduced repolarization heterogeneity was maintained for 6 months; (4) VA and major adverse events occurred less frequently in the HBP and control groups than in the RVP group; and (5) Patients with VA demonstrated longer QRS duration, QT interval, Tpe, and Tpe/QT ratio at 6 months than those without VA during the follow‐up period.

RVP causes longer QRS durations and prolonged QT intervals associated with decreased cardiac function and VA occurrence (Cho et al., 2016; Del‐Carpio Munoz et al., 2017). Since the QT interval comprises the beginning of ventricular depolarization to the end of repolarization, a longer QT interval is related to depolarization and repolarization instability and malignant VA development. In contrast, physiological pacing, HBP is unlikely to be affected by these malignancy concerns, which is supported by our finding that the QRS duration, Tpe, and Tpe/QT ratio were significantly shorter in the HBP group than in the RVP group postoperatively. Although there may be a period of electrical remodeling and adaptation to the new abnormal activation sequence during RVP (Wecke et al., 2007), left ventricular remodeling due to RVP might increase repolarization heterogeneity thereafter (Cvijic et al., 2018). The slightly decreased LVEF after 6 months and desynchrony in the RVP group of the present study might be early signs of ventricular remodeling that is possibly linked to further LVEF deterioration and VA events thereafter.

A previous study demonstrated that left bundle branch area pacing was superior to RVP in reducing repolarization markers on ECG assessed at 48 h postoperatively (Wang et al., 2020). They reported that left bundle branch area pacing yielded a narrower paced QRS duration, shorter QT and QTc interval, lower QT dispersion, and shorter Tpe than RV septal pacing in the acute phase after pacemaker implantation. Recent studies demonstrated the significant benefits of repolarization parameters on ECG after conduction system pacing compared with those after cardiac resynchronization therapy at a mild‐term assessment period of up to 6 weeks or a median of 42 days following implantation (Gupta & Pavri, 2022; Sarkar et al., 2022). In contrast to previous studies, we implemented a sufficient follow‐up period of 6 months to reduce the effect of ventricular repolarization instability during the acute phase and elicited a continuous advantage of repolarization stability after HBP in the chronic phase. Physiological pacing, HBP, and left bundle branch area pacing have the potential advantage of reducing the effect of ventricular repolarization instability versus non‐physiological pacing. Moreover, the benefit of repolarization stability may additionally impact the greater recovery of cardiac function from HBP in patients with HF compared with that from cardiac resynchronization therapy (Hua et al., 2022; Kato et al., 2022; Upadhyay et al., 2019). However, this hypothesis requires more research.

Tpe interval and Tpe/QT ratio are ECG markers of ventricular repolarization that were recently proposed to predict VA events and sudden cardiac death in individuals with cardiac diseases and the general population (Tse et al., 2017, 2018). The Tpe interval in the precordial leads reflects the transmural axis of the left ventricle as an index of transmural dispersion of repolarization. Endocardial pacing, even RVP, suppresses the transmural dispersion of repolarization earlier than epicardial bi‐ventricular pacing, with a reduced risk of VA (Marrus et al., 2012; Wecke et al., 2005). However, in the chronic phase after RVP, ventricular remodeling and desynchronization emerge after non‐physiological pacing. These negative effects might be linked to repolarization instability and increased VA risk that could not be observed in physiological pacing (Itoh et al., 2013; Marrus et al., 2012). In contrast, JT interval, another repolarization indicator, was not a significant predictor of outcomes in this study (Zhou et al., 1992). Although the exact reason for this is unclear, the Tpe interval is a more sensitive marker of repolarization abnormality than the JT interval that may be potentially influenced by several patient conditions of heart rate and autonomic nervous system (Hnatkova et al., 2017; Kusuki et al., 2021).

Our findings also indicate that the repolarization indices were almost identical between the HBP and control groups, although the QRS duration was shorter in the control versus HBP group. The results of the present study imply similar repolarization stability and minimal difference in ventricular myocardium stimulation between HBP and the native ventricular conduction system.

### 4.1 Study limitations

This was a single‐center retrospective small‐sample study. The population was heterogeneous among study groups, which may have influenced the outcomes and changes in ECG parameters. Specifically, the cardiac function and baseline characteristics of the HBP and RVP groups differed somewhat, and distinct changes in the ECG parameters should have been evaluated under identical conditions before device implantation. RVP was a secondary option after HBP failure in some patients in the RVP group, which might carry bias of severe condition. Such a population might have more damage and fibrosis in the right atrium and conduction system behind the HBP failure. The higher mean BNP value and longer QRS duration and QT interval at baseline in the RVP group also support the abovementioned hypothesis. These confounding factors that could potentially influence the ECG changes should be acknowledged in this small‐sample size analysis. Repolarization parameters are affected by the patient's condition, autonomic nervous system, intrinsic conduction morphology, and temporal pacing before implantation due to abrupt changes to different activation sequences (Rosenbaum et al., 1982). It is unclear whether VA events newly developed after pacemaker implantation or occurred due to their original underlying disease with the risk of VA augmentation. Nonetheless, this pilot study can address an important physiological question regarding depolarization/repolarization dynamics with physiological versus non‐physiological pacing and its relationship to VA risk. Future studies on larger populations with organized pacing protocols and patient settings are required.

## 5 CONCLUSIONS

HBP showed better depolarization and repolarization stability than RVP and was similar to native ventricular conduction, which might be associated with a reduced risk of VA occurrence. HBP has the potential ability to reduce the effect of ventricular repolarization instability compared to non‐physiological pacing.

## AUTHOR CONTRIBUTION

S.Y designed the study, analyzed the data and wrote the manuscript. Y.I. helped the data interpretation. S.Y., Y. I., R.W., N.T., N.S., T.N., M.S., T.O., S.R., K.F., and K.S. carried out the examination. R.S., and M. T. supervised this work.

## CONFLICT OF INTEREST

Drs. Yanagisawa and Shibata are affiliated with a department sponsored by Medtronic Japan. The other authors declare no conflicts of interest.

## ETHICAL APPROVAL

This study was approved by the local institutional ethics committee of Nagoya University Hospital. All patients provided written informed consent prior to undergoing the procedure.

## Data Availability

The data that support the findings of this study are available from the corresponding author upon reasonable request.
